# Atomistic understanding of self-healing of a ferroelastic crystal

**DOI:** 10.1039/d5sc06790a

**Published:** 2025-11-05

**Authors:** Zarif Fahim, Patrick Commins, Liang Li, Panče Naumov, Qiang Zhu

**Affiliations:** a Department of Mechanical Engineering and Engineering Science, University of North Carolina at Charlotte Charlotte NC USA qzhu8@charlotte.edu; b Smart Materials Lab, New York University Abu Dhabi PO Box 129188 Abu Dhabi United Arab Emirates pance.naumov@nyu.edu; c Department of Sciences and Engineering, Sorbonne University Abu Dhabi PO Box 38044 Abu Dhabi United Arab Emirates; d Center for Smart Engineering Materials, New York University Abu Dhabi PO Box 129188 Abu Dhabi United Arab Emirates; e North Carolina Battery Complexity, Autonomous Vehicle and Electrification (BATT CAVE) Research Center Charlotte NC 28223 USA

## Abstract

The recent discovery of the self-healing capabilities of molecular crystals has shown significant efficiency, approaching nearly 100%, particularly when this process is coupled with a phase transition. This places these materials on par with other, better-studied soft materials, such as polymers. However, the physical inaccessibility of the contact interface with common analytical methods hinders direct experimental observation of the critical molecular-scale processes responsible for the recovery of the interfacial gap; as a result, this effect has largely remained phenomenological. This report employs molecular dynamics simulations and mechanical analysis to unravel the mechanistic details behind the remarkably efficient (95%) self-healing observed in the ferroelastic crystal anilinium bromide. Bulk simulations successfully reproduce the experimentally observed phase transitions under both uniaxial and biaxial loading conditions, while slab model calculations with free surfaces capture crack formation and self-healing phenomena associated with twinning and detwinning. The atomistic insights establish a two-step model: external mechanical loading first activates molecular slip along the (110)[11̄0] path, providing a periodic impulse force that encourages molecular reorientation, leading to twinning and detwinning, along with subsequent ferroelastic and self-healing behaviors. These findings underscore the critical roles of crystal packing and mechanical loading and offer clear design principles for developing new organic self-healing materials with enhanced mechanical properties.

## Abstract 2

The recent discovery of the self-healing capabilities of molecular crystals has shown significant efficiency, approaching nearly 100%, particularly when this process is coupled with a phase transition.

## Graphical abstract

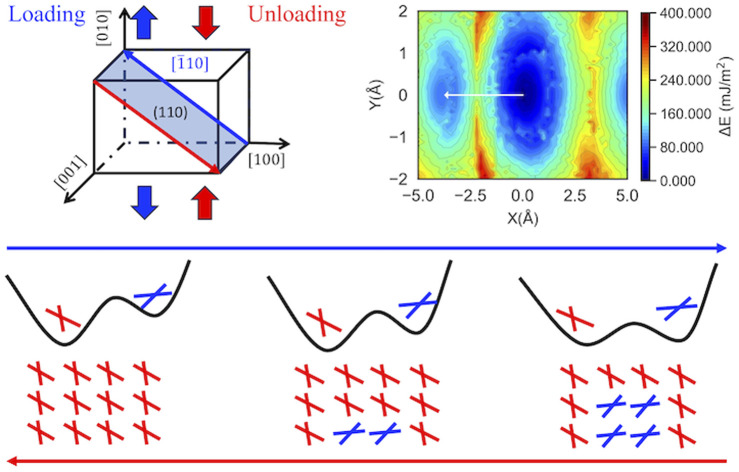

## Introduction

The ability of some materials to repair themselves over time after they have been damaged is a fascinating phenomenon, both from a fundamental perspective and with compelling analogies with living organisms, as well as from the view of the enormous practical implications it could have for devices that operate over long time or that are not accessible for direct control due to remote location or extreme environments.^1–5^ Self-healing has reached the level of commercial appeal in paints,^6,7^ thin films,^8–10^ and polymers.^11–14^ The reformation of bonds that drives self-healing, which can occur *via* both diffusive- and non-diffusive processes, requires transport of matter and sufficient molecular dynamics to reform interactions (covalent, coordination, or intermolecular) at the temperature at which it occurs;^15–17^ it is thus common for soft materials, where the molecules are relatively free and flexible. Autonomous repair is unanticipated for crystalline materials, where the three-dimensional order may imply slower diffusion and therefore, decreased propensity for structural recovery. Recent examples, however, have demonstrated healing in molecular crystals, with yields that range from less than 10% for dynamic covalent bond formation^18^ to about 95% when this process is coupled to a strain-induced ferrolastic phase transition, observed with crystals of anilinium bromide (AniHBr).^19^ This efficacy of self-healing during phase transitions appears to be superior to that based on other processes.^20–24^ Specifically, ferroelastic crystals possess multiple stable orientational states that can be reversibly switched when external stress exceeds a critical threshold, and this characteristic often manifests alongside shape-memory effects and superelasticity, as exemplified by the archetypal shape-memory material nitinol.^25^ The exotic properties have enabled ferroelastic materials to find widespread applications ranging from piezoelectric sensors to mechanical switches.^26–29^ Historically, research on ferroelastic phase transitions has predominantly focused on inorganic compounds,^30^ however, these materials often suffer from limited flexibility, which can hinder their practical applications. In contrast with inorganics, pure organic ferroelastic and hybrid inorganic–organic crystals offer distinct advantages, most notably, the ability to undergo large-angle deformation.^31–45^ With the ever-increasing library of organic ferroelastic materials, they have superseded the inorganic materials in their energy dissipation capabilities.

Although the occurrence of self-healing is undisputed, one of the greatest challenges in understanding this phenomenon is the lack of experimental information on the molecular-scale processes that occur at the interface between the two fragments. Direct observation is naturally limited by the inaccessibility of contact-based methods; only the unhealed surface is available for inspection, for example, through dye-staining and confocal-microscopic observation. Surface microscopic methods provide only indirect information, such as mass migration, while optical methods yield only partial insights, typically related to diffusion-related processes or the recovery of crystallinity. None of the currently available non-destructive methods provide direct information on the bond-reformation process, and this limitation hampers a thorough investigation of the self-healing process. To overcome this challenge, molecular dynamics (MD) simulations become valuable in the study of self-healing by offering atomistic insights directly from the analysis of molecular trajectory.^46–50^ However, MD simulations of organic crystals are generally not very popular in the community due to the nontrivial simulation setup and force field availability. Recently, we have developed an automated pipeline to enable rapid computational screening of organic crystals with flexible mechanical properties.^51^ Using this pipeline, we aim to investigate the ferroelastic and self-healing properties of AniHBr, and understand the fundamental mechanisms underlying these transitions. Here, we will present our simulation results of structural transformations under various loading conditions, as well as the atomistic pathways extracted from both the simulated phase transitions and healing processes. Our analysis implicates a microscopic two-step mechanism, where external mechanical load first activates molecular sliding along the (110)[−110] path, which then provides a periodic impulse force to encourage molecular reorientation leading to twinning and detwinning. The insights gained from this study will provide a deeper understanding of the phase transition mechanisms in AniHBr and suggest design principles for developing new organic ferroelastic materials with enhanced mechanical properties.

## Computational methods

### Crystal structure

**Fig. 1:**
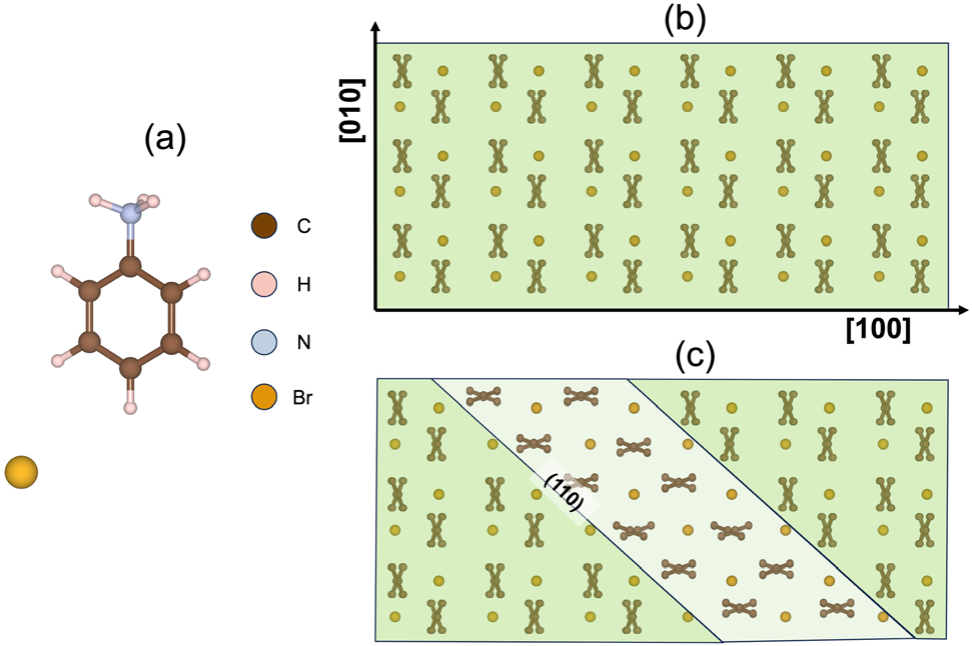
crystal structure of *P*2_1_/*c* AniHBr. (a) Illustration of a single aniline cation and a bromide anion. (b) The supercell of the pristine structure viewed along the (001) plane. (c) Supercell with a partially formed twin domain, also viewed along the (001) projection. The X-shaped motif, formed by two adjacent layers of aniline molecules, is used to highlight the twinning behavior.

To date, AniHBr is known to exhibit two distinct solid forms near room temperature.^52,53^ Among these two forms, the monoclinic phase has been well studied due to the ferroelastic behavior.^19,53,54^ It was initially assigned to the *P*2_1_/*c* symmetry (CSD code: ANLINB02)^52^ and then reassigned to the *P*2_1_/*m* symmetry (CSD code: ANLINB09, ANLINB10 and ANLINB12) with two mixed aniline orientations in the recent studies.^19,53^ Computationally, the *P*2_1_/*c* representation can be considered as an orientationally ordered version of the *P*2_1_/*m* phase along the crystallographic *c*-axis. Hence, we used ANILIB02 in *P*2_1_/*c* symmetry to construct our model and avoid the partial occupation issue. In this *P*2_1_/*c* phase, each unit cell comprises four aniline cations and four bromide anions. For clarity, a single aniline cation and a bromide anion are depicted in Fig. 1a. The (001) projection is shown Fig. 1b. From this projection, one can clearly see that two aniline cations are stacked with alternative inclination angles and they form the vertically aligned X-shaped pattern. Our recent experiment^19^ reported that a fraction of X-shaped aniline molecules, upon external mechanical loading, may undergo a primary rotation along the [001] axis to locally form a twin domain featured as the horizontally stacked X-shaped pattern along the (110) plane of the parent phase, as depicted in Fig. 1c. Upon the release of mechanical load, the twinning domain may undergo a detwinning process to return to the pristine state, thus resembling a ferroelastic phase transition.

### Model setup

To mimic the experimentally observed ferroelastic phase transition, we used two different types of models in our calculation: (i) bulk model, periodic boundary conditions in every direction, used to calculate the reversible ferroelastic phase transition and (ii) slab model, periodic boundary conditions in the *xy* plane along with non-periodic conditions in the *z* axis, used to model the crack formation and self-healing.

For the bulk model, we set a simulation box of 326 × 448 × 34 Å^3^ that contains 442 368 atoms. For the slab model, a simulation box of 432 × 34 × 919 Å^3^ (423 936 atoms) was used. In this slab model, a vacuum region of 600 Å was added along the *z*-axis to create non-periodic boundary conditions and avoid interactions across periodic images. In addition, the structure in the slab model was rotated to align the *z*-axis along the [100] direction and the *x*-axis along the [010] direction for non-periodic boundary conditions along the *z*-axis to mimic the experimental setup.^19^ Note that in the slab setting, only an *xy* tilt (no *xz* or *yz*) is allowed. Hence, one has to ensure the *xy* plane to be close so as to be orthogonal to the *z*-axis for the general cases of monoclinic and triclinic crystals. However, this is not the case for *P*2_1_/*c*-AniHBr since its cell parameters are already close to an orthogonal box.

After the models were constructed, we investigated them by using the LAMMPS package^55^ with the choice of the particle–particle particle-mesh solver.^56^ Here, we employed a pipeline^51,57^ to automate force field parameter generation based on the OpenFF 2.0.0 (sage) toolkit,^58,59^ with atomic charges using the semi-empirical AM1-BCC method.^60^ To assess the reliability of generated force field parameters, we equilibrated the systems at 273 K under constant number–pressure–temperature (NPT) conditions and calculated the lattice parameters, as shown in Table 1. The calculated values show good agreement with experimental results, confirming the validity of our force field choice.

**Table 1:** The comparison of cell parameters for the *P*2_1_/*c* AniHBr phase at 273 K from experiments and MD simulations

Source	*a* (Å)	*b* (Å)	*c* (Å)	*α* (°)	*β* (°)	*γ* (°)
Expt.^52^	6.758	5.981	16.738	90.00	91.34	90.00
OpenFF	6.796	6.197	16.945	90.03	90.01	90.01

### Direct MD simulation of cycling loads

During the simulation, the systems are initially equilibrated at the given temperature for 100 ps under the NPT ensemble with 1 fs per timestep. Two different calculations are done on the bulk model: (i) uniaxial tension/compression along [010] and (ii) bi-axial compression along [100] and [010] directions alternatively. For the uniaxial load on [010], we applied a maximum tensile strain of 15%, and then released it to the original states *via* compression. This calculation is done with two different strain rates. Initially, a high strain rate of 1 × 10^−7^ fs^−1^ is applied until 10% of the total strain (corresponding to 1 ns simulation per 10% strain change) during tension and rest of the calculation is done with a much lower strain rate of 5 × 10^−9^ fs^−1^ (corresponding to 20 ns simulation per 10% strain change). After we found that the strain rate did not significantly impact the results, we adopted a fixed strain rate of 1 × 10^−7^ fs^−1^ for the remaining calculations. For the bi-axial calculation, we compressed along [100] initially and then compressed along [010] with a maximal compressive strain of 14% on [100] and a tension of 14% on [010]. Another similar uniaxial deformation calculation is done on the slab model with a maximal [010] strain of 12%. In all simulations, we employed the Nose–Hoover thermostat and barostat to control the temperature and pressure, respectively.^61^ In addition, molecular rotation has been found to play a crucial role in understanding the phase transition process. To analyze molecular orientation changes during the MD simulation, we used the tracking and analysis scripts established in our previous work.^51,57^

### Molecular slide simulations

Molecular slip has been found to play an essential role in generating mechanical flexibility and phase transitions. Following our recent work,^57^ we performed the γ-surface energy calculation, a widely used concept for metal deformation, to evaluate the spatial distribution of penalty energy required for a crystal to slide along a particular plane. To compute the γ-surface energy, we employed a systematic approach where a chosen slip plane was displaced incrementally while relaxing the surrounding lattice. For each displacement, the total energy of the system was calculated using LAMMPS, capturing the energy landscape associated with partial or complete shifts of the crystal along the slip plane.

Using the γ-surface results, we performed more detailed slip calculation along the selected path to understand the molecular twinning and detwinning mechanisms. To mimic the sliding motion, we progressively shifted the upper grain along the (110)[−110] direction to simulate molecular sliding. After each sliding step, we allowed the system to relax for 10 ps to reach a new equilibrium state. To achieve better control over the simulation, we constrained both top and bottom layers of the slab model to move as rigid bodies, while allowing only the four middle layers to move freely. Each layer contains 192 molecules. Initially, the middle four layers were equilibrated at 300 K. After each sliding step, we equilibrated the system under the constant number–volume–temperature (NVT) ensemble for 10 ps at 300 K to reach a new equilibrium state. This approach aimed to study the molecular behavior around the slide plane where molecules are connected to the bulk on either side.

## Results and discussion

In this section, we present the results of atomistic modeling of AniHBr's deformation *via* MD simulations. We will first discuss deformation under three different loading conditions, including (i) uniaxial tensile–compression cycle on the bulk, (ii) biaxial compressions on the bulk, and (iii) uniaxial tensile–compression cycle on the slab model. Finally, the mechanistic insights and the connection with experimental observation, as well as the impact of simulation parameters, will be thoroughly discussed.

### Bulk deformation under uniaxial cycling loads

**Fig. 2:**
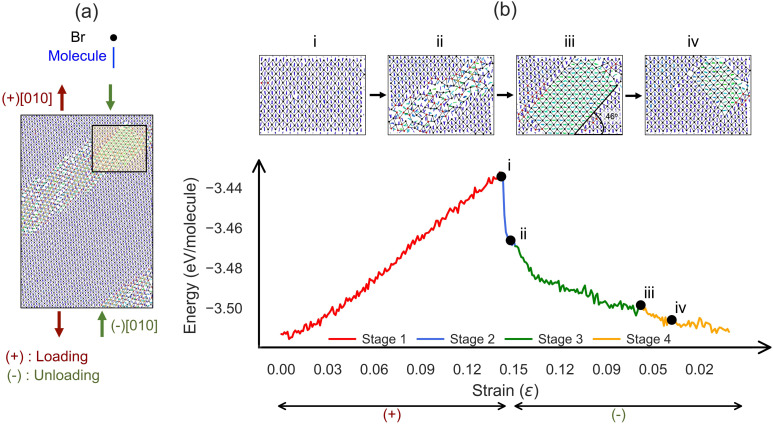
MD simulation results for the uniaxial bulk tension/compression cycle along the [010] direction at 350 K. (a) A section of the simulated structure, highlighting a region with pronounced molecular reorientation. (b) The energy evolution as a function of strain (lower panel, for a total of 41 ns MD simulation), with four representative MD snapshots (i–iv) shown above. In all structure plots, molecules are colored by their rotation around the [001] axis relative to the initial orientation, and bromine atoms are colored by coordination number: black (4-fold), red (5-fold), and yellow (6-fold).

We first applied uniaxial tension and compression along the [010] direction of AniHBr using a periodic model. To clarify the discussion, we use + and – superscripts to denote whether the strain conditions are in loading or unloading stages, respectively. Fig. 2a displays the atomic configuration of the entire supercell under 5^−^% compressive strain, revealing the formation of distinctive structural domains separated by well-defined boundaries. Upon loading, these domains exhibit different coordination environments and molecular reorientations. The yellow-boxed region in Fig. 2a highlights an area exhibiting significant structural transformation that will be the focus of our detailed analysis in the following discussion.

Fig. 2b and SI movie S1 both show the energy evolution as a function of loading strain within the one complete tension–compression cycle. To elucidate the underlying atomistic mechanism, we selected four representative snapshots at different strain loads from the energy profile shown in Fig. 2b(i–iv) as enlarged views of the yellow-boxed deformation region in Fig. 2a. For clarity, only one Br layer and two alternating aniline layers are displayed. The Br atoms are colored black (4-coordinated), red (5-coordinated), and yellow (6-coordinated) within a cutoff distance of 5.9 Å, while the aniline molecules are colored based on their rotational displacement from the initial configuration. From the energy profile in Fig. 2b, we can divide the whole cyclic process into four distinct stages based on their structural features and strain conditions.

• Stage 1: elastic deformation under [010] tension (0 → 14^+^%). Under tensile loading, the system exhibits linear elastic behavior with a quadratic increase in energy relative to strain. During this stage, the structure retains its crystalline order while gradually deforming until reaching the elastic limit at approximately 14^+^% strain. In the meantime, the X-shaped molecular arrangement gradually changes as the angle between adjacent aniline rings approaches 0°, resulting in vertical alignment up to the elastic limit; see Fig. 2b(i). Throughout this process, the Br anions retain their original 4-coordination environment.

• Stage 2: formation of a defect stripe with a rapid energy drop (14^+^% → 13^−^%). Beyond the elastic deformation limit, the system exhibits a sudden energy drop indicating an abrupt structural transition. As shown in Fig. 2b(ii), this transition involves formation of a deformed stripe region along the (110) plane. Within this region, Br atoms transition from 4- to predominantly 5- or 6-coordination, while the aniline molecules undergo significant disordered rotations. Upon the backward compression, the system's energy continues to reduce while more Br atoms adopt 6-coordination. The deformation stripe expands in width during this process. According to our visual analysis, this stage roughly ends around 13^−^%, though its transient nature may make experimental observation challenging.

• Stage 3: twinning formation (13^−^% → 6^−^%). Following the defect formation, the system's energy continues to decrease but with a reduced slope, which corresponds to the twinning formation. With more compression, the twinned nucleus expands along the deformation stripe, eventually forming a large twinning domain as shown in Fig. 2b(iii) (*ε* = 7.1^−^%) spanning multiple molecular layers. The calculated angle between the twinning domain and the bulk is 46°, matching well with 48° as found in the experiment.^19^

• Stage 4: detwinning under [010] compression (6^−^% → 0^−^%). Under further compression, the twinning domain progressively shrinks as displayed in Fig. 2b(iv) (approximately a strain of 4-%). Different from the twinning formation, the detwinning stage appears to be achieved without the disordering states for both Br and aniline rings. This process continues with the unloading and the twin domain nearly disappears at zero strain. In this process, the energy continues to decrease and the system ultimately returns to a configuration closely resembling the initial state.

While the above discussion is focused on the partial region marked in Fig. 2a, it is important to note that the entire supercell undergoes similar structural transformations. A similar plot illustrating the full simulation domain is provided in the SI (Fig. S1–S3). In general, the aforementioned four stages can be observed even when we vary the simulation parameters (*e.g.*, the strain rate, maximum strain and temperature), suggesting that this is a robust phenomenon. Compared with the experimental findings, our simulation successfully reproduces several key features, including (i) the formation of well-defined twin boundaries at about 48°, (ii) nearly complete recovery upon unloading, and (iii) molecular motions mimicking the twinning and detwinning. The close agreement between simulation and experiment encouraged us to further explore the system's response under different loading conditions.

### Bulk deformation under bi-axial compressive loads

**Fig. 3:**
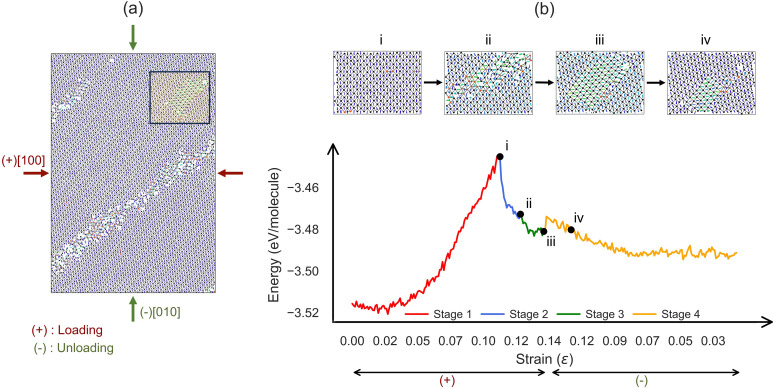
MD simulation results for the biaxial bulk compression cycle along the [100] and [010] directions at 350 K. (a) A section of the simulated structure, highlighting a region with pronounced molecular reorientation. (b) The energy evolution as a function of strain (lower panel, for a total of 2.8 ns MD simulation), with four representative MD snapshots (i–iv) shown above. In all structure plots, molecules are colored by their rotation around the [001] axis relative to the initial orientation, and bromine atoms are colored by coordination number: black (4-fold), red (5-fold), and yellow (6-fold).

In the experiment,^19^ it was found that ferroelastic transition in AniHBr can also be realized in a bi-axial compressive loading. To mimic this finding, we performed a bi-axial loading simulation on the bulk model. The system was first compressed along the [100] direction followed by compression along the [010] direction. The energy profile and structural evolution are shown in Fig. 3 and SI Fig. S4–S6 and movie S2. Using the same approach from the previous section, we focused on the part marked in Fig. 3a, and plot four representative snapshots of this domain in Fig. 3b(i–iv), corresponding to four stages as follows:

• Stage 1: elastic deformation under [100] compression (0 → 10.8^+^%). Similar to uniaxial tension, the system's energy increases quadratically with strain. The elastic deformation reaches its limit at approximately 10.8% compressive strain as shown in Fig. 3b(i).

• Stage 2: formation of defect stripes (10.8^+^% → 12.0^+^%). Further compression triggers a phase transition, marked by a significant drop in energy. As shown in Fig. 3b(ii), formation of a defect stripe corresponds to the phase transition along the (110) plane, accompanying the emergence of 5/6 coordination of Br atoms and molecular rotation disordering in this defect region.

• Stage 3: twinning formation (12.0^+^% → 14.0^+^%). As compression continues, the energy decreases to reach a minimum described by a twinned domain (see Fig. 3b(iii)).

• Stage 4: detwinning at [010] compression (14.0^−^% → 0^−^%). When the [010] compression is applied, the system follows a gradual energy decrease, accompanied by the molecular detwinning as shown in Fig. 3b(iv). However, the whole system cannot fully recover to the original state, due to residual deformation in other regions.

Compared with the uniaxial tension–compression loading cycle, our bi-axial compressive loading reveals a similar 4-stage behavior but it triggers the phase transition more efficiently, and allows the twinning formation during the loading stage. These observations are consistent with experimental findings where the twinning was found to accompany the loading stage,^19^ except that this loading type tends to be more destructive and thus can create more permanent damage to the system. Consequently, some of the deformed regions did not recover their original configuration, in contrast to the bulk uniaxial calculation, indicating permanent damage. Given that MD simulations are inherently limited by time scales, the partial detwinning observed in our simulations may be due to the limited simulation time.

### Slab deformation under uniaxial tensile–compressive load

**Fig. 4:**
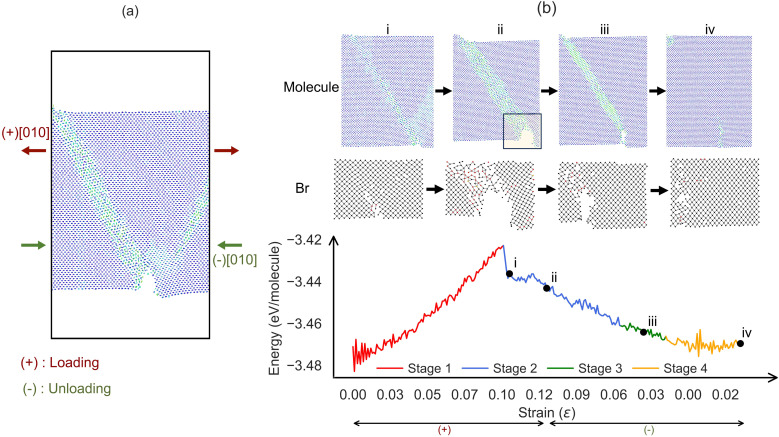
MD simulation results for the slab model under uniaxial tensile–compressive loading along the [010] direction at 350 K. (a) The simulated slab model. (b) The lower panel shows the simulated energy profile under the uniaxial tension/compression cycle loading along [010] on the slab model at 350 K (for a total of 2.6 ns MD simulation). The upper panel shows four representative MD snapshots with molecule and bromine views, respectively. The bromine atoms are colored by coordination number: black (4-fold), red (5-fold), and yellow (6-fold).

The aforementioned bulk modeling, under full periodic boundary conditions, is only suitable for understanding the mechanical tests for the ideal bulk system. Hence, these settings may introduce the artificial periodic interaction to suppress the motions that occur near the edge of the sample, *e.g.*, the formation of a crack near the tip under the mechanical loads. To truly mimic the experimental cracking formation and self-healing process,^19^ we employed a slab model as shown in Fig. 4a (consisting of free surfaces along the *z*-axis) by applying a uniaxial [010] tension/compression load up to a maximum of 12% tensile strain. The energy profile for this slab model under cycle loading is shown in Fig. 4b and SI movie S3. Similar to previous analysis, we divide the whole process into four stages as follows:

• Stage 1: elastic deformation under [010] tension (0 → 10.4^+^%). Under tensile loading, the system exhibits elastic behavior, until it reaches the limit as shown in Fig. 4b(i).

• Stage 2: formation of defect stripes and cracks (10.4^+^% → 6^−^%). Beyond the elastic limit, significant energy is found to be released, indicating a phase transition. The deformation stripe is then formed along the (110) plane. Due to the existence of free surface, it becomes clear that molecular sliding along the (110) plane creates the deformation. This motion not only triggers changes in Br's coordination and molecular orientation, but also creates a crack at the slab's lower surface (see Fig. 4b(ii)). The crack then continues to expand and propagate as the strain increases to 12.0^+^%. Upon backward compression, the right half of molecules is forced to slip upwards along the (110) plane.

• Stage 3: twinning (6.0^−^% → 2.2^−^%). As the system is compressed further to *ε* = 6.0^−^%, some molecules start to form the twinned nucleus at multiple locations along the deformation stripe, as shown in Fig. 4b(iii). As compression continues, the twinned domain expands and grows larger in size. In the twinned domain, the Br atoms in this region revert to their 4-coordination environment, and the aniline molecules become ordered again, which is similar to the twinning formation in the bulk model. Accompanying the twinning formation, the crack size is reduced further.

• Stage 4: detwinning and healing of the crack (2.2^−^% → −2.0^−^%). When *ε* becomes smaller than 2.2^−^%, the twinned domain starts to detwin (see Fig. 4b(iv)) and the crack is progressively closed, until the system is healed.

In general, the slab model exhibits a notably smaller elastic strain limit of 10^+^% compared to 14^+^% in the bulk model. This difference can be attributed to surface effects in the slab model, which significantly influence the material's mechanical behavior.

Furthermore, the slab simulations successfully capture the key features of the self-healing process observed experimentally. Using a single MD run, we observed both crack formation and subsequent self-healing – a phenomenon rarely captured in previous organic crystal modeling work. Importantly, our simulations clearly reveal that molecular sliding along the (110) plane is the primary mechanism triggering the formation of defect stripes and subsequent twinning/detwinning processes. In addition, the formation of twinned domains along these defect stripes and their subsequent detwinning appears to reduce the crack size, effectively healing the material back to its original state.

### Effects of temperature

**Fig. 5:**
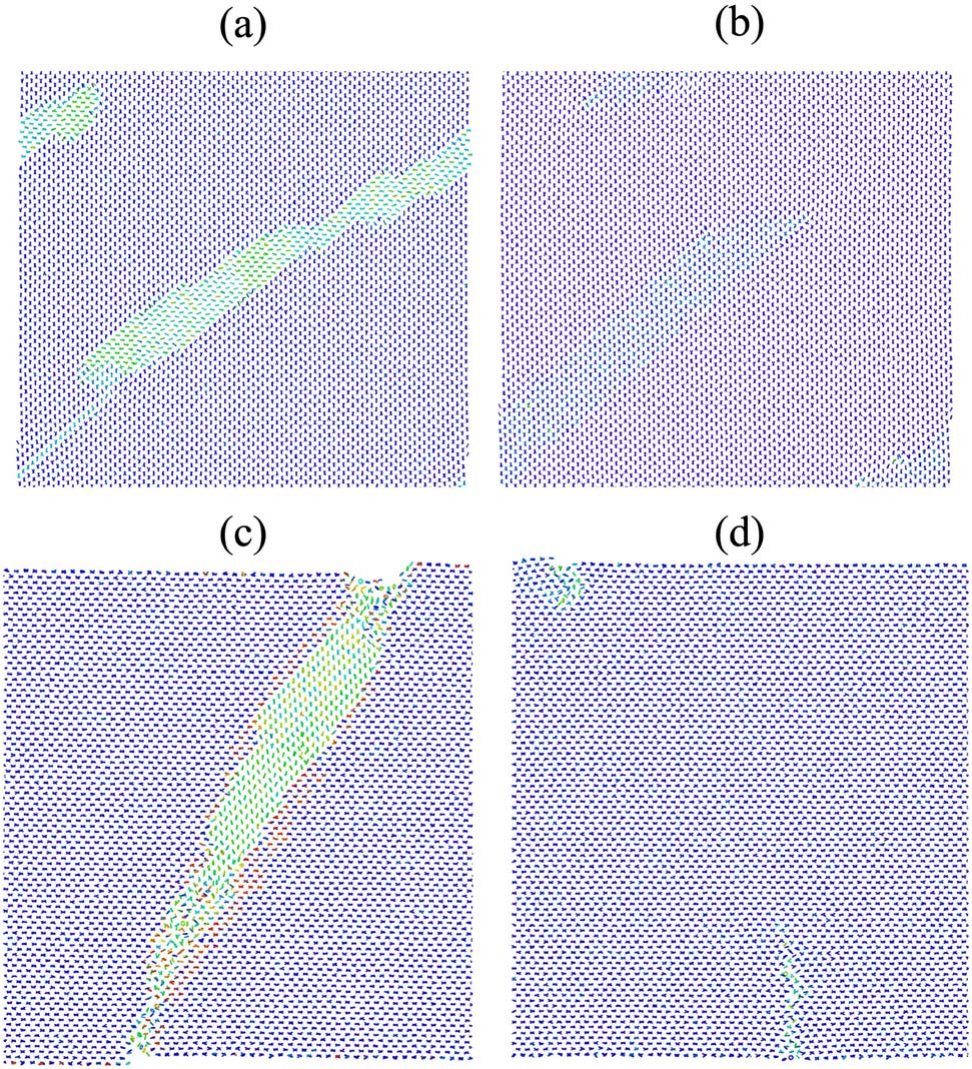
Comparison of final MD configurations at 300 K and 350 K. The final states at the end of the bulk calculation cycle are shown at (a) 300 K and (b) 350 K. For the slab calculation, the final states of the system are shown at (c) 300 K and (d) 350 K. For clarity, the Br cations are omitted, and the aniline molecules are colored based on the molecular rotation along the [100] axis.

It is important to note that all previously discussed results were based on MD simulations at 350 K, which differs from the room temperature conditions used in the actual experiments.^19,53,62^ For comparison, we also performed MD simulations at 300 K. The results are summarized in Table 2 and Fig. 5.

**Table 2:** Comparison between different MD simulations of the ferroelastic behavior in AniHBr

Model	Temperature (K)	Loading type	Stage 1 (%)	Stage 2 (%)	Stage 3 (%)	Stage 4 (%)
			Elastic deformation	Defect stripe at (110)	Twinning	Detwinning
Bulk	300	Uniaxial	[0^+^, 9.8^+^]	[9.8^+^, 7.5^−^]	[7.5^+^, 4.5^−^]	[4.5^−^, 0^−^]*
Bulk	300	Biaxial	[0^+^, 10.8^+^]	[10.8^+^, 3.0^−^]	[3.0^−^, 0^−^]	N/A
Slab	300	Uniaxial	[0^+^, 9.6^+^]	[9.6^+^, 7.2^−^]	[7.2^−^, – 2.0^−^]	N/A
Bulk	350	Uniaxial	[0^+^, 14.0^+^]	[14.0^+^, 13.0^−^]	[13.0^−^, 6.0^−^]	[6.0^−^, 0^−^]
Bulk	350	Biaxial	[0^+^, 10.8^+^]	[10.8^+^, 12.0^+^]	[12.0^+^, 14.0^+^]	[14.0^+^, 0^−^]
Slab	350	Uniaxial	[0^+^, 10.4^+^]	[10.4^+^, 6.0^−^]	[6.0^−^, 2.2^−^]	[2.2^−^, – 2.0^−^]

Fig. 5a and c show the final configurations for bulk and slab calculations, respectively, at 300 K. In both cases, twinned domains were observed during the unloading stage and persisted after further unloading. While partial detwinning occurred in some locations of the simulation models for the bulk uniaxial calculation, complete detwinning was never observed at 300 K. In addition, neither bulk biaxial compression cycle nor slab uniaxial cycle calculations exhibited any evidence of detwinning. Specifically, in the slab model, additional compression along the [010] direction could not trigger the detwinning process.

In contrast, Fig. 5b and d show the corresponding results at 350 K. The twinned domains formed more readily during both loading and unloading stages, and could fully detwin to return to the original crystalline state. This indicates that both twinning and detwinning are more likely to occur at elevated temperatures, as the additional thermal energy helps overcome the energy barriers to achieve the structural ordering for twinning and detwinning.

Table 2 provides a comprehensive comparison between our MD simulations and experimental observations of ferroelastic behavior in AniHBr. Partial detwinning and no detwinning at 300 K can be attributed to the high strain rate applied in these MD simulations. At a high strain rate, the molecules do not have sufficient time to dissipate energy, preventing the transition from a high energy state to a low energy one. However, it is impossible to reach an experimental stain rate in the MD simulation due to the limitation of time scale. Hence, we increased the temperature to overcome the shortcomings of high strain rate. Atomic mobility increases and the barrier to low energy also becomes smaller. Across different simulation parameters, the four-stage transition model remains consistent. However, the duration of each stage varies significantly with simulation conditions. These discrepancies and inconsistencies suggest that our direct MD simulations, while providing valuable insights into the atomic-level mechanisms, are inherently limited by the time scales involved, as well as the model setup.

### Molecular sliding analysis

To gain deeper insight into the microscopic mechanism underlying twinning and detwinning, we revisit our MD results from an atomistic perspective. Across all simulations, these processes are consistently associated with the formation of defect stripes and molecular slip along the (110) plane. This observation suggests a two-step mechanism: (i) external mechanical loading activates molecular sliding on the (110) plane and (ii) the resulting sliding motion induces twinning and detwinning transitions. By decomposing the process into these two steps, we can better analyze the limitations of direct MD simulations and refine our understanding of the experimentally observed twinning/detwinning phenomena. We therefore proceed to systematically test the validity of this two-step hypothesis.

**Fig. 6:**
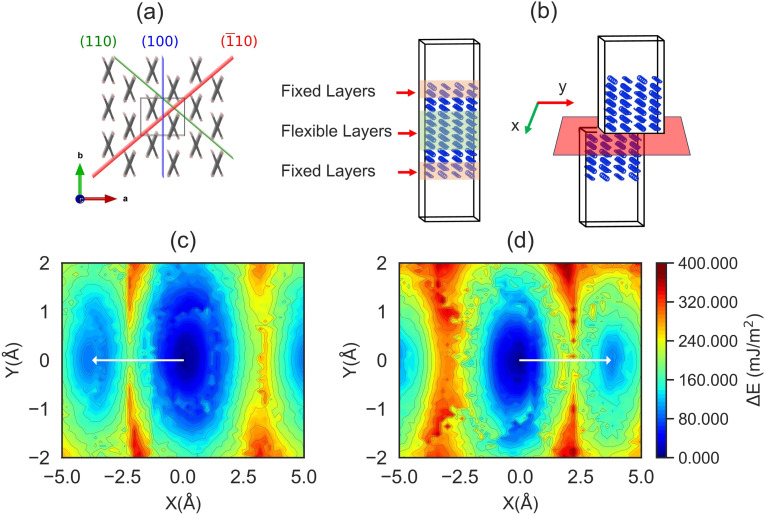
Molecular sliding in the AniHBr crystal. (a) The available slip planes identified from crystal packing analysis. (b) The schematic setup for MD-based slip calculations. (c and d) The calculated γ-surface energy profiles for the (110) and (1̄10) planes at 0 K, respectively.

First, we clarify the conditions required for molecular sliding. From the molecular packing analysis (see Fig. 6a), the AniHBr crystal possesses three planes with interlayer spacings that are plausible for slip: 1.486 Å in (100), and 0.173 Å in both (110) and (11̄0). Among these planes, the (001) plane is unlikely to serve as a slip plane due to unfavorable alignment with respect to experimental [010] tension or [100] compression. Instead, these loads can effectively generate a sizable partial force component to trigger molecular sliding along the (110) or (11̄0) planes.

Using the slip calculation setup in Fig. 6b, our γ-surface calculations (shown in Fig. 6c and d) confirm that both planes have very low activation energy barriers for molecular sliding. In particular, the (110)[1̄10] and (11̄0)[110] slip systems exhibit an exceptionally low activation energy barrier of 153.1 mJ m^−2^, which is similar to recent studies.^50,57,63^ While our direct MD simulations show that sliding events were triggered by global elastic deformation, such large-scale deformation may not be necessary in real experiments. In solid mechanics, it is well established that uniaxial tension or compression can activate slip systems before significant structural transformations due to the presence of crystal defects like vacancies or dislocations. Compared to metals, slip systems in organic crystals are more readily accessible under small strains due to weaker intermolecular interactions. Therefore, our direct MD simulations based on ideal crystal arrangements likely overestimate the strain required to activate the slip system.

**Fig. 7:**
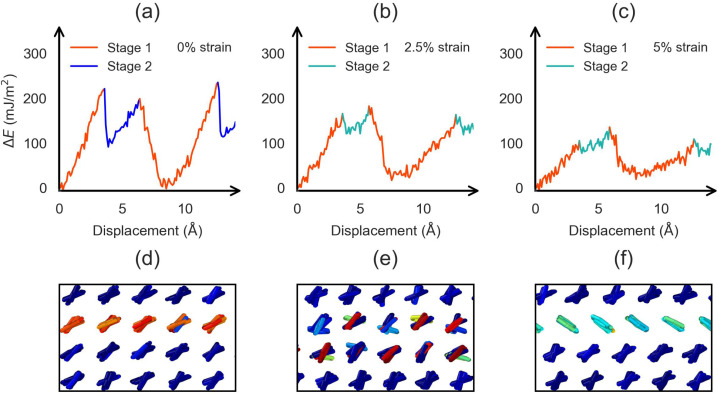
The simulated molecular sliding along the (110)[110] slip path under varying strain conditions at 300 K. (a–c) The simulated periodic impulse potential profiles as a function of displacement along the (110)[110] slip path at different strain levels. (d–f) Representative molecular configurations corresponding to the right-aligned (R), partially flipped, and fully flipped orientation states, respectively.

Second, we investigated if the (110)[1̄10] slip can trigger the twinning and detwinning processes. The sliding simulation results are summarized in Fig. 7 and SI movie S4. The sliding along the (110)[1̄10] direction at zero strain (Fig. 7a) periodically induces molecular flipping in the central layers of aniline molecules. By analyzing the molecular alignment, each period can be divided into two regions, highlighted in red and blue in Fig. 7a. The red region corresponds to molecules with a right-aligned configuration and lower average energy (Fig. 7d), while the blue region corresponds to partially left-aligned configuration with higher average energy (Fig. 7e). Thus, continuous sliding provides a periodic impulse that promotes reorientation of the aniline molecules. Nevertheless, it is important to note that sliding along the (110) plane alone does not result in stable twinning, as the molecules tend to revert to their original orientation once the impulse is removed. This suggests that the original orientation is energetically preferred under zero strain. As such, no twinning is observed in the sliding simulation at zero strain.

However, the left-aligned molecular states become energetically favorable when mechanical strain is applied. As illustrated in Fig. 7b and c, sliding under tensile strains of 2.5% and 5.0% not only facilitates the transition to this orientation but also stabilizes the left-aligned configuration of the aniline molecules. As shown in Fig. 7f, the molecules can be completely flipped to left-aligned states with a lower average energy. This indicates that the left-aligned configuration is energetically preferred when the system is under tensile strain. Furthermore, we investigated whether our sliding simulation results depend on the simulation cell size. As shown in Fig. S7 of the SI, varying the cell size does not lead to any qualitative change in the results.

### A sliding-mediated microscopic twinning model

**Fig. 8:**
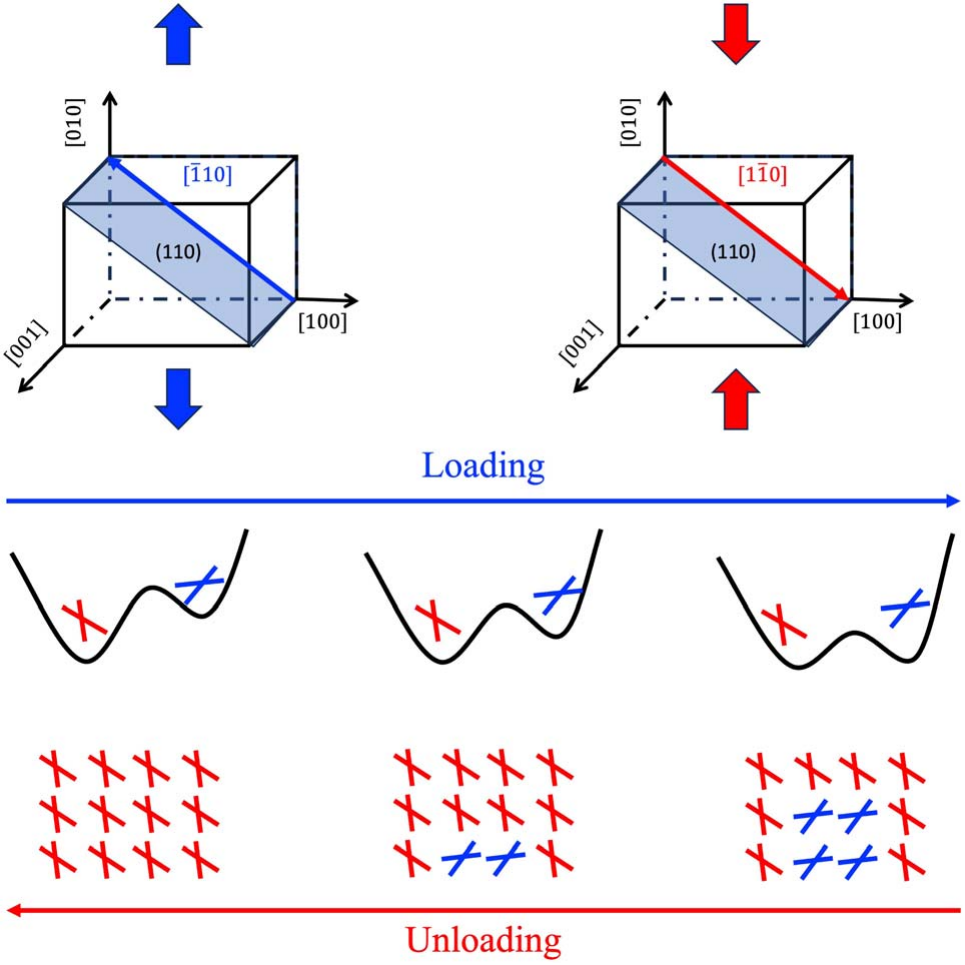
The proposed microscopic twinning/detwinning mechanism according to the MD simulation.

From a thermodynamic perspective, an external strain modifies the potential energy landscape, lowering the energy barrier and making the alternative orientation state increasingly accessible. As the strain increases, the energetic preference for the left-aligned state becomes more pronounced, allowing a larger fraction of molecules to adopt this configuration. This strain-induced stabilization of the alternative orientation is a key factor in the nucleation and growth of twinned domains observed during the ferroelastic phase transition. Thus, the interplay between mechanical loading and molecular sliding provides a microscopic pathway for the reversible switching between orientation states, which underlies the observed twinning and detwinning phenomena in AniHBr. Based on these observations, we propose the microscopic mechanism illustrated schematically in Fig. 8.

First, external mechanical loading—such as [010] tension or [100] compression—activates the (110)[1̄10] slip system, generating a periodic impulse that facilitates reorientation of the aniline molecules.

Second, the applied strain modifies the energy landscape of aniline orientations, making the left-aligned state energetically favorable under tension. The periodic impulse generated by sliding facilitates this reorientation, promoting the accumulation of left-aligned molecules and the nucleation and growth of twinned domains. Upon unloading, the energetic preference shifts back to the original orientation, and sliding then encourages molecules to revert to their initial state, resulting in detwinning.

Structurally speaking, these processes are governed by molecular sliding along the (110) plane, which leads to the experimentally observed twinning and detwinning domains. This mechanism is consistent with the measured 46° twin boundary angle.

## Conclusions

In this work, we investigated the ferroelastic and self-healing behaviors of AniHBr. Our direct MD simulations, employing both bulk and slab models, successfully reproduce the experimentally observed ferroelastic transitions under uniaxial and biaxial loading, as well as self-healing phenomena. The phase transition proceeds through four distinct stages: (i) elastic deformation, (ii) formation of defect stripes, (iii) twinning nucleation and growth, and (iv) detwinning upon unloading.

Using the atomistic insights extracted from our MD simulations, we propose a two-step microscopic model for twinning and detwinning in AniHBr. In this mechanism, external mechanical loading first activates molecular sliding along the (110) plane, generating a periodic impulse that facilitates the reorientation of aniline molecules. This reorientation leads to twinning or detwinning, depending on the relative energetic stability of the orientation states, which is modulated by the applied strain. Our hypothesis is supported by crystal packing analysis and targeted sliding simulations using slab models.

Our molecular simulations quantitatively reveal the critical roles of crystal packing and mechanical loading in governing ferroelastic and self-healing behaviors. Two key factors are identified for optimizing these properties:

Crystal packing: the slip plane should ideally have a layer spacing between 0 and 0.5 Å. If the spacing is too small, the activation barrier becomes prohibitively high; if too large, the impulse force may be insufficient to trigger the molecular reorientation required for ferroelastic transition. For AniHBr, the (110) plane has a spacing of 0.17 Å, enabling efficient slip activation.

Mechanical loading: mechanical strain plays a dual role by (i) activating the slip system and (ii) tuning the energetic preference between orientation states (twinning/detwinning). This highlights the potential for strain engineering to optimize ferroelastic transitions through precise control of loading conditions.

These insights provide clear design principles for developing next-generation organic ferroelastic materials with enhanced mechanical characteristics.

## Author contributions

Q. Z. and P. N. co-conceived the idea and supervised this project. Z. F. performed the MD simulations. All authors (Z. F., P. C., L. L., P. N. and Q. Z.) analyzed the results and contributed to manuscript writing.

## Conflicts of interest

There are no conflicts to declare.

## Supplementary Material

SC-OLF-D5SC06790A-s001

SC-OLF-D5SC06790A-s002

SC-OLF-D5SC06790A-s003

SC-OLF-D5SC06790A-s004

SC-OLF-D5SC06790A-s005

## Data Availability

The source code, instructions, as well as scripts used in this study, are available at https://github.com/zfahim57/AniHBr-2025. Supplementary information is available. See DOI: https://doi.org/10.1039/d5sc06790a.
