# Supplementary material for: Atomistic understanding of self-healing of a ferroelastic crystal
Source: Chem Sci. 2025 Nov 5;17(1):335–45. doi: 10.1039/d5sc06790a (PMC12604052; doi:10.1039/d5sc06790a)
Supplement: SC-017-D5SC06790A-s001 [file SC-017-D5SC06790A-s001.pdf]

Supplementary Information (SI) for Chemical Science.
This journal is © The Royal Society of Chemistry 2025

Supplementary Online Materials: Atomistic Understanding of Ferroelastic Phase Transition and Self-Healing in Aniline-HBr

Zarif Fahim,[1] Patrick Commins,[2] Liang Li,[3] Pance Naumov,[2,4] and Qiang Zhu[1,5]

[1)] *Department of Mechanical Engineering and Engineering Science, University of North Carolina at Charlotte, Charlotte, NC 28223, USA*

[2)] *Smart Materials Lab, New York University Abu Dhabi, PO Box 129188, Abu Dhabi, UAE*

[3)] *Department of Sciences and Engineering, Sorbonne University Abu Dhabi, PO Box 38044, Abu Dhabi, UAE*

[4)] *Center for Smart Engineering Materials, New York University Abu Dhabi, PO Box 129188, Abu Dhabi, UAE*

[5)] *North Carolina Battery Complexity, Autonomous Vehicle and Electrification (BATT CAVE) Research Center, Charlotte, NC 28223, USA*

(Dated: 8 November 2025)

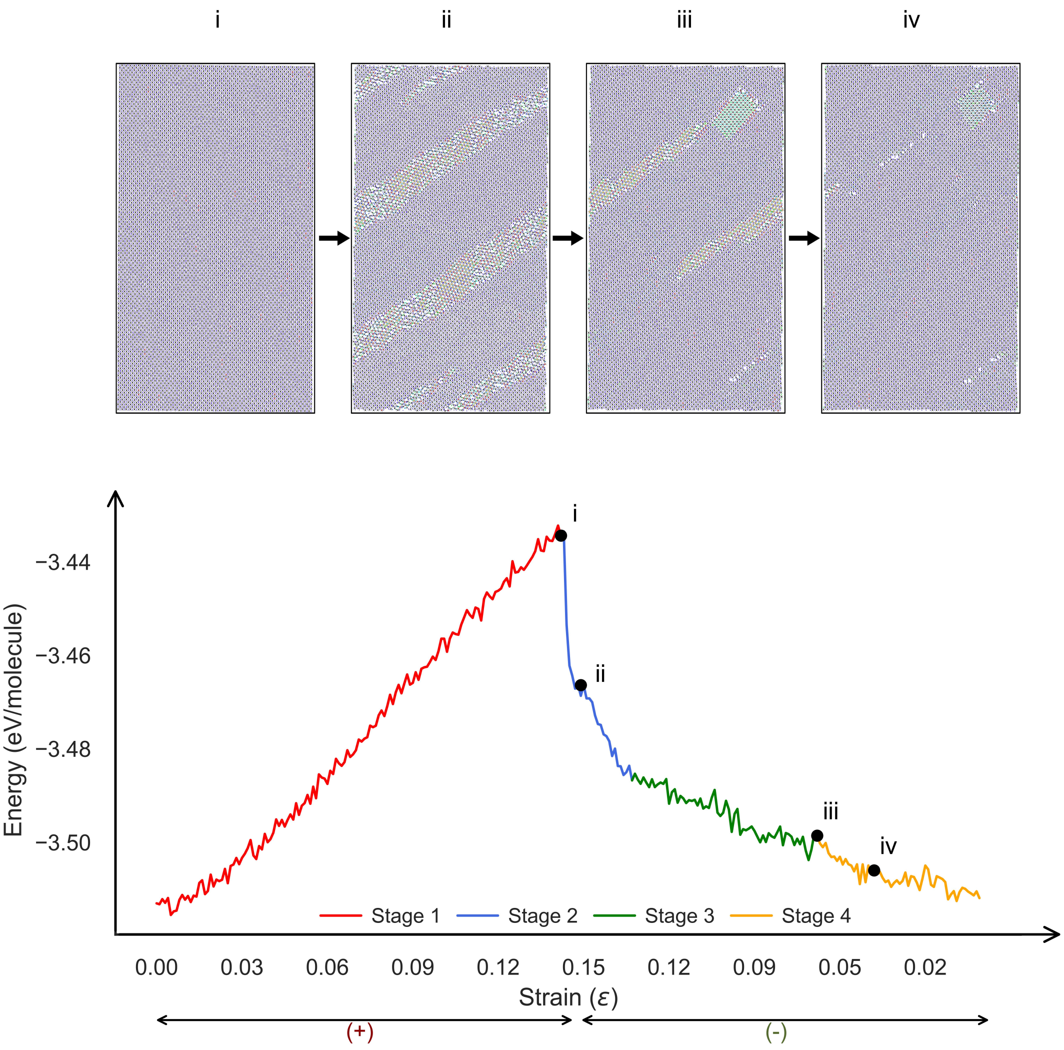

FIG. S1. MD simulation results for the uniaxial bulk tension/compression cycle along the [010] direction at 350 K. The energy evolution as a function of strain (lower panel), with four representative MD snapshots of the entire simulation domain (i–iv) shown above. In all structure plots, molecules are colored by their rotation around the [001] axis relative to the initial orientation, and bromine atoms are colored by coordination number: black (4-fold), red (5-fold), and yellow (6-fold).

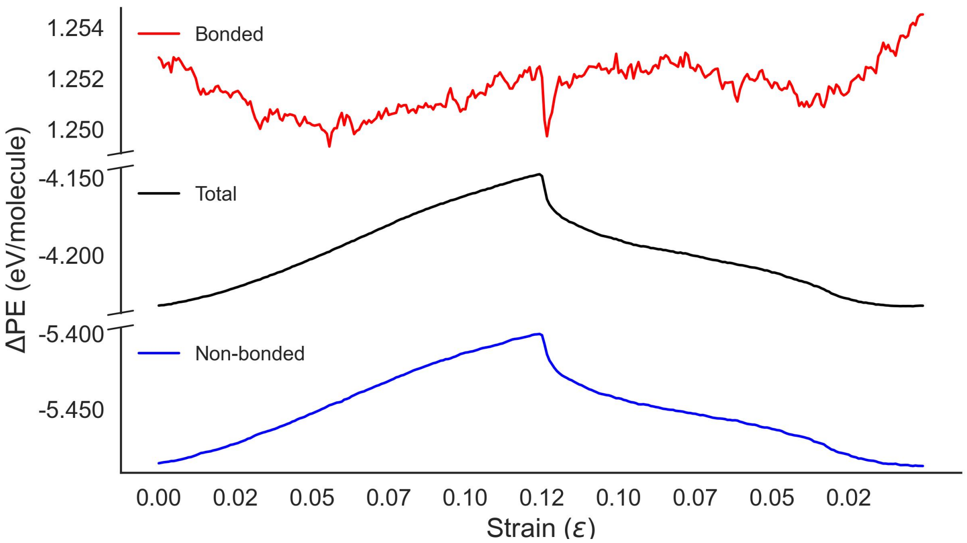

FIG. S2. The progress of potential energies during the MD for uniaxial bulk tension/compression cycle along the [010] direction at 350 K.

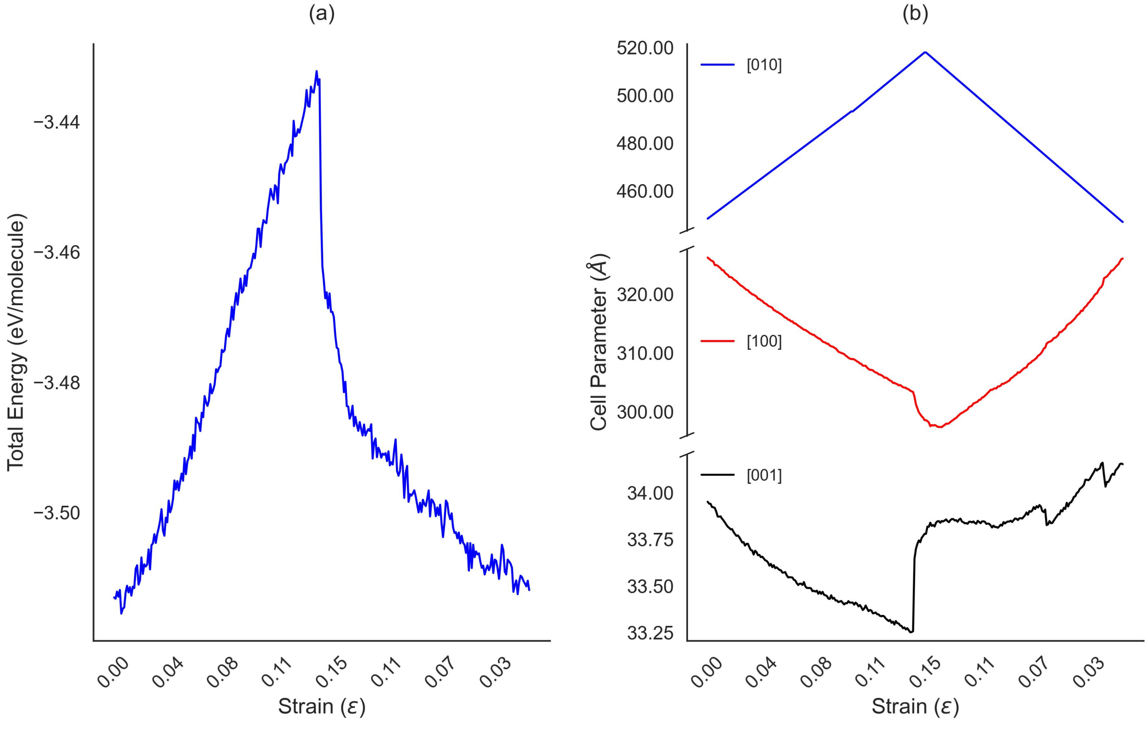

FIG. S3. The progress of (a) total energy and (b) cell dimensions during the MD for the uniaxial bulk tension/compression cycle along the [010] direction at 350 K.

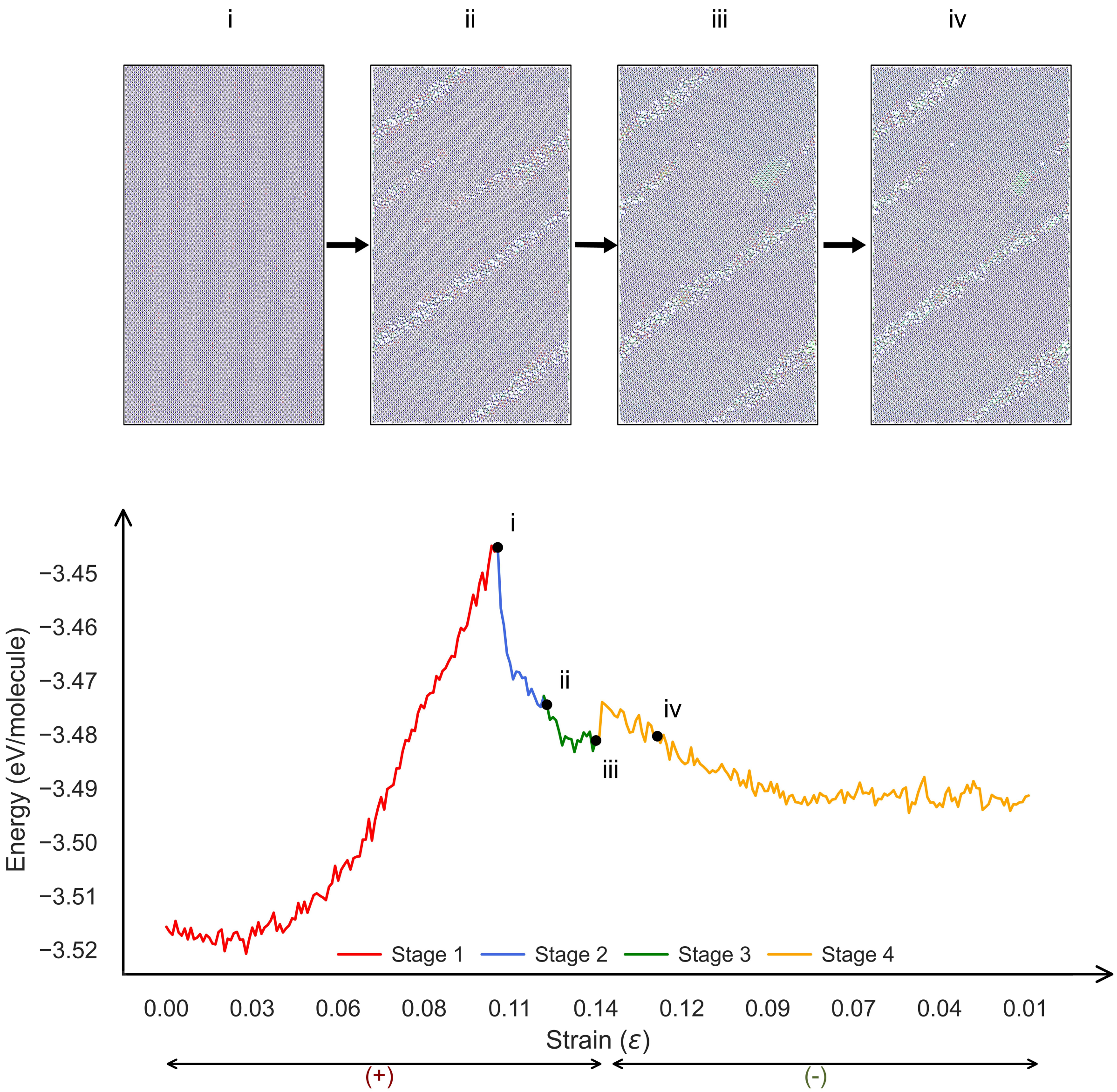

FIG. S4. MD simulation results for the biaxial bulk compression cycle along the [100] and [010] directions at 350 K. Presents the energy evolution as a function of strain (lower panel), with four representative MD snapshots (i–iv) shown above. In all structure plots, molecules are colored by their rotation around the [001] axis relative to the initial orientation, and bromine atoms are colored by coordination number: black (4-fold), red (5-fold), and yellow (6-fold).

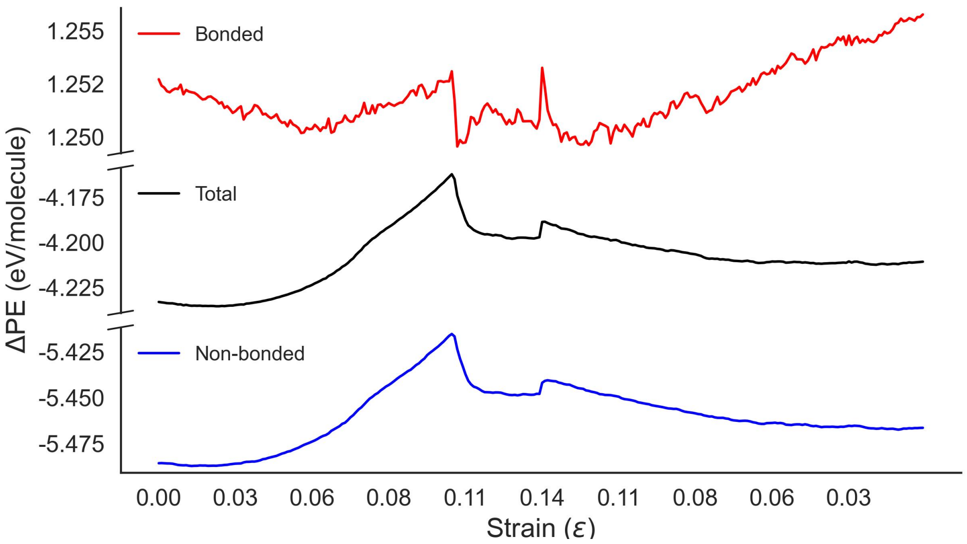

FIG. S5. The progress of potential energies during the MD for biaxial bulk compression cycle along the [100] and [010] directions at 350 K.

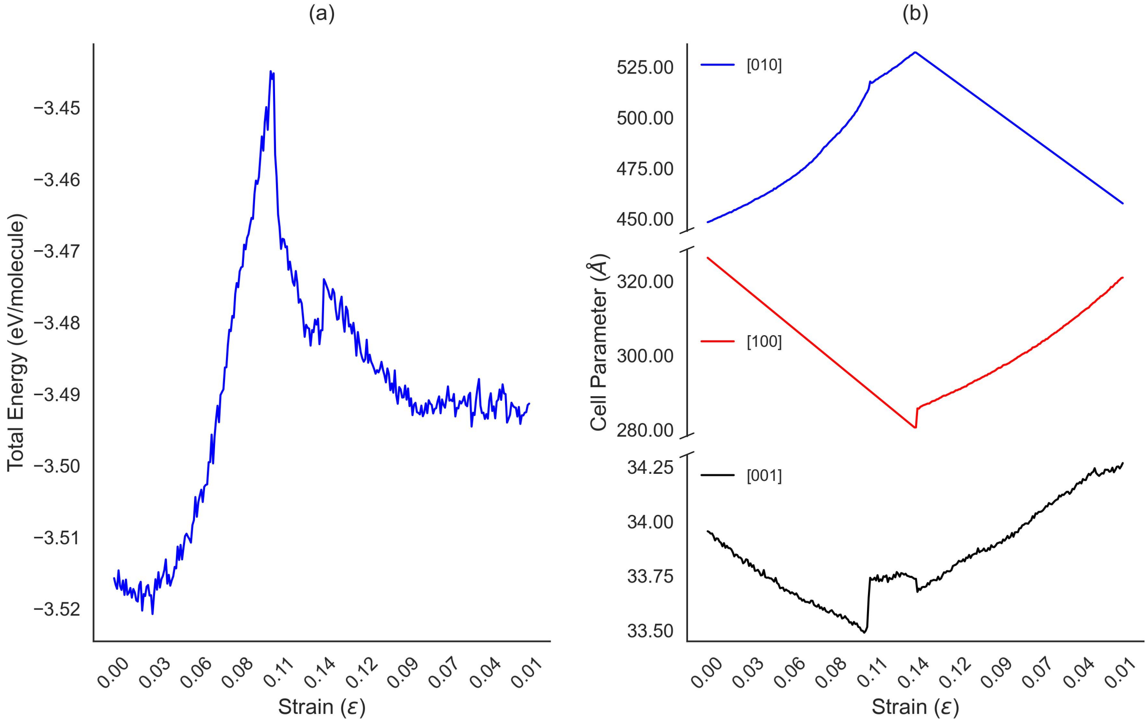

FIG. S6. The progress of (a) total energy and (b) cell dimensions during the MD for biaxial bulk compression cycle along the [100] and [010] directions at 350 K.

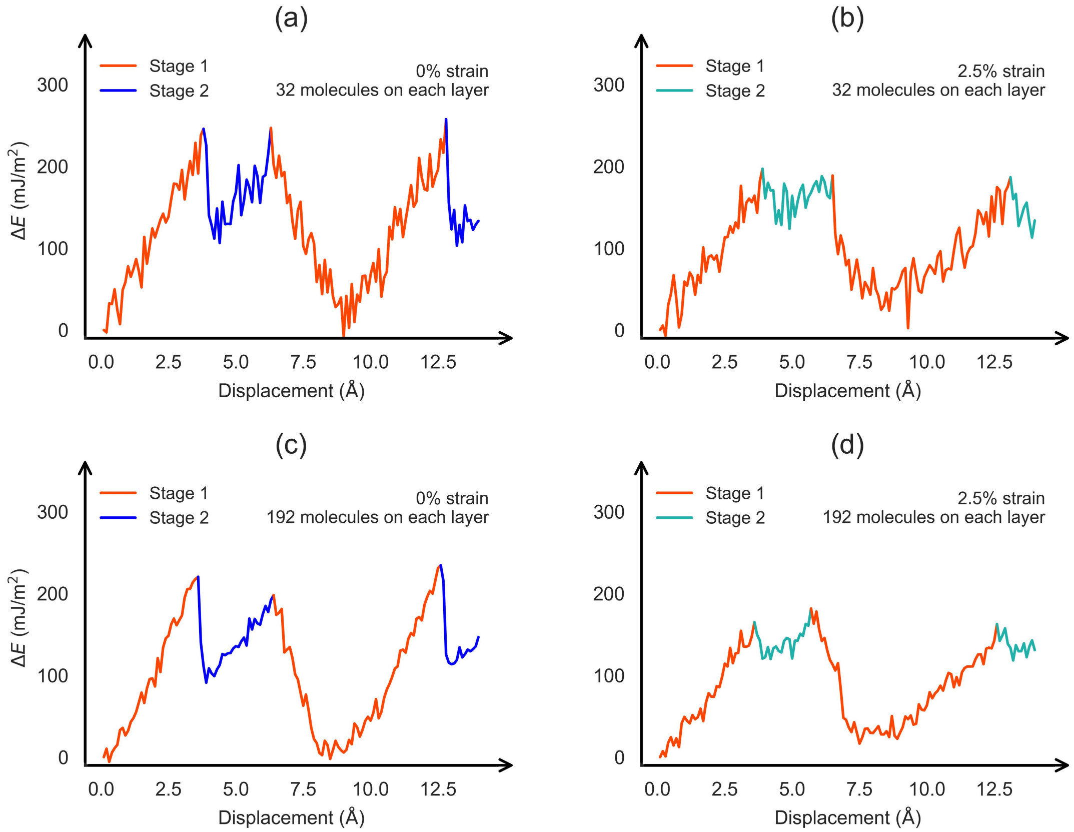

FIG. S7. Size dependence of molecular sliding calculation. (a) and (b) show the results for 32 molecules per layer; while (c) and (d) show the results for 192 molecules per layer. The minor differences suggest that the results are not sensitive to the choice of unit cell sizes.
